# Blockade of CXCL12/CXCR4 signaling inhibits intrahepatic cholangiocarcinoma progression and metastasis via inactivation of canonical Wnt pathway

**DOI:** 10.1186/s13046-014-0103-8

**Published:** 2014-12-04

**Authors:** Shengqiang Zhao, Jing Wang, Chengyong Qin

**Affiliations:** Department of Gastroenterology, Shandong Provincial Hospital, Shandong University, Jinan, China

**Keywords:** Intrahepatic cholangiocarcinoma (IHCC), CXC chemokine ligand-12 (CXCL12)/chemokine receptor type 4 (CXCR4), Prognosis, Metastasis, Wnt pathway

## Abstract

**Background:**

Intrahepatic cholangiocarcinoma (IHCC) is the second most frequent primary malignant liver tumor following hepatocellular carcinoma. It is a highly fatal disease and has few therapeutics. The CXC chemokine ligand-12 (CXCL12)/CXC chemokine receptor type 4 (CXCR4) axis has been shown to be involved in tumorgenesis, proliferation, and angiogenesis in a variety of cancers including IHCC. However, its prognostic significance in IHCC is unclear. The purpose of this study was to examine the functional role of CXCR4 in the progression and metastasis of IHCC and explore the underlying mechanism.

**Methods:**

The CXCR4 expression, overall survival, and the clinical characteristics including age, sex, differentiation degree, tumor size, vascular invasion, lymph node metastasis, TNM stage, and T stage were analyzed for 122 IHCC patients. Short hairpin RNA (shRNA) against CXCR4 was used to disrupt the CXCL12/CXCR4 signal transduction pathways in IHCC cell lines. *In vitro* assays, including CCK-8 assay, flow cytometry, and colony formation assay, and *in vivo* tumor formation assay were utilized to detect the cell phenotype of CXCR4 knockdown cells. Transwell and wound healing assays were used to examine the IHCC cell invasion and migration ability. The Wnt pathway was assessed by Western blot and β-Catenin/Tcf transcription reporter assay.

**Results:**

We demonstrated that CXCR4 expression was closely correlated with IHCC progression and metastasis characteristics. The overall survival of patients with high CXCR4 expression was significantly lower than that of patients with low CXCR4 expression. Furthermore, we showed that the abrogation of CXCR4 had significantly negative influence on the IHCC cell phenotype, including *in vitro* cell proliferation, cell cycle, colony formation, cell invasion, and *in vivo* tumorigenicity. In addition, CXCR4 knockdown downregulated Wnt target genes and mesenchymal markers such as Vimentin and Slug.

**Conclusions:**

In conclusion, our result shows that high CXCR4 expression is associated with IHCC progression and metastasis via the canonical Wnt pathway, suggesting that CXCR4 may serve as a promising therapeutic target for IHCC.

## Background

Intrahepatic cholangiocarcinoma (IHCC) is a malignancy whose pathogenesis involves abnormal biliary epithelial differentiation [1]. It is the most frequent primary malignant liver tumor next to hepatocellular carcinoma and is highly fatal because of its early invasion, widespread metastasis, and the lack of an effective therapy [2,3]. Therefore, it is urgent to uncover the molecular mechanisms of IHCC and identify potential therapeutic targets to improve the treatment. Chemokine receptors form a large family of proteins that mediate chemotaxis of cells towards a gradient of chemokines. Many studies have shown that chemokines and their receptors are implicated in the development of different types of cancers [4-6]. One of the best studied chemokine receptors is CXCR4. CXCR4 is a G protein-coupled chemokine receptor, encoded on chromosome 2 [7]. During embryonic development, CXCR4 is expressed on progenitor cells, allowing the migration from their birthplace to their final destination where they will differentiate into organs and tissues. In the late 1990s, CXCR4 expressed on CD4+ T cells was found to serve as a co-entry receptor for human immunodeficiency virus HIV-1 [8]. The following-up studies also found that CXCR4 can mediate the metastasis of a variety of cancers [4,6,9,10]. CXCR4 selectively binds the CXC chemokine ligand-12 (CXCL12, or SDF-1), which has been found to be important in the tumorigenesis, proliferation, metastasis, and angiogenesis in cancers [11,12]. CXCR4 has been reported to be upregulated in more than 20 cancers, including ovarian [13], prostate [14], esophageal [15], melanoma [16], neuroblastoma [17], and renal cell carcinoma [18], and plays an important role in the communication of cancer cells with their microenvironment [19,20]. Moreover, CXCR4-positive cancer cells can migrate toward distant organs in response to CXCL12 gradient. By inhibition of CXCR4, the growth and invasion of cancer cells can be impaired [21-23]. In 2014, T. Yu et al. [24] found that suppressing expression of CXCR4 by MicroRNA-9 could inhibit the proliferation of oral squamous cell carcinoma cells both *in vitro* and *in vivo* through the Wnt/β-catenin signaling pathway, and activation of CXCR4 expression led to the constitutive activation of β-catenin, implying the important role of Wnt/β-catenin in CXCR4 signaling, which was consistent with the previous reports in colorectal cancer [25], ovarian cancer [26], pancreatic cancer [23], and bone marrow stromal cells [27].

In cholangiocarcinoma, Ohira et al. [28] demonstrated that CXCR4 was mainly expressed in IHCC cells and CXCL12 in stromal fibroblasts, and the interaction of CXCL12 released from fibroblasts and CXCR4 expressed on IHCC cells may be actively involved in IHCC migration, suggesting CXCR4 could be a therapeutic target to prevent IHCC invasion. This possibility was confirmed by Gentilini et al. [29] using AMD3100, a non-peptide antagonist of the CXCR4, and Tan et al. [30] using siRNA targeting at CXCR4. In 2012, CXCL12/CXCR4 was further reported to mediate angiotensin II-enhanced epithelial-to-mesenchymal transition (EMT) in IHCC [31]. More recently, Leelawat K. et al. [4] found that CD24 could induce CXCR4 expression in cholangiocarcimoma cells, which may assist invasion of the cancer cells. When treated by AMD3100, the motility and invasiveness of CD24 (+) cells were decreased, implying the importance of CXCR4 in cholangiocarcinoma cell invasion. However, the precise function of CXCR4 and the signal transduction pathways following CXCR4 activation in IHCC remain elusive. The aim of this study was to define the role of CXCR4 in IHCC and elucidate the underlying mechanism.

## Methods

### Cell culture

Human intrahepatic cholangiocarcinoma cell lines, HuCCT1 (ATCC, Manassas, VA, USA), HCCC-9810 ( Keygen Biotech, China), RBE ( Keygen Biotech, China), and Huh28 (Keygen Biotech, China) were cultured at 37°C in RPMI 1640 medium (Hyclone) supplemented with 10% fetal calf serum, 100 U/ml penicillin, and 100 mg/ml streptomycin in humidified atmosphere containing 5% CO_2_.

### Immunohistochemistry

Samples including 122 primary IHCCs, 75 matched metastatic lymph nodes, and 122 adjacent non-cancerous liver tissues containing normal intrahepatic bile ducts (at least 5 cm distant from the tumor edge) were obtained from the Department of Pathology, Shandong Provincial Hospital. Immunohistochemical staining for CXCR4 was performed using the SABC kit (Boster, Wuhan, China) according to the manufacturer’s instruction. Primary antibody for CXCR4 (1:50, polyclonal, Abcam, MA, USA) was used for overnight incubation at 4°C. For the evaluation of CXCR4 IHC staining, a semi-quantitative scoring criterion was used, in which both the staining intensity and positive areas were recorded. A staining index (values 0–12), obtained as the intensity of CXCR4-positive staining (weak, 1; moderate, 2; strong, 3) and the proportion of immune-positive cells of interest (0%, 0; <10%, 1; 10–50%, 2; 51–80%, 3; >80%, 4), were calculated. The cases were grouped into low (score 0–6) and high (scores 8–12) CXCR4 expression. The study was approved by the Ethics Committee of Shandong Provincial Hospital Affiliated to Shandong University, as stipulated by the Declaration of Helsinki, with written informed consent for the use of the specimens from all enrolled patients.

### Construction and transfection of CXCR4 shRNAs

This study utilized three CXCR4 shRNA targeting different regions of the CXCR4 [GenBank: NM_003467]. The shCXCR4-1 targeted CXCR4 mRNA at nucleotides 1093-1111 with sense: 5′- AGCGAGGTGGAC ATTCATC-3′, and antisense: 5′- GATGAATGTCCACCTCGCT -3′; The shCXCR4-2 targeted CXCR4 mRNA at nucleotides 741-759 with sense: 5′- CTGTCCTGCTATTGCATTA -3′, and antisense: 5′- TGACAGGACGACGATAACGTAAT -3′; The shCXCR4-3 was designed to be homologous to nucleotides 206-224 of the human CXCR4 with sense: 5′-TGAGAAGCATGACGGACAA-3′, antisense: 5′-TTGTCCGTCATGCTTCTCA-3′ [23]. A negative control, targeting at no region in human genome, was designed with sense: 5′-TTCTCCGAACGTGTCACGT-3′, antisense: 5′-ACGTGACACGTTCGGAGAA-3′. These shRNA oligos were cloned to lentiviral vector pLKO.1 following the instruction provided by Addgene (Boston, MA, USA). All constructs were verified by sequencing. Stable transfections were performed using Lipofectamine 2000 (Invitrogen, Carlsbad, CA, USA) according to the manufacturer’s instructions.

### Quantitative real-time RT-PCR

RNA was isolated from cells and reverse-transcribed. Real-time RT-PCR Primers specific for target genes were as follows: CXCR4, forward 5′-GATCAGCATCGACTCCTTCA-3′ and reverse 5′-GGCTCCAAGGAAAGCATAGA-3′; β-catenin, forward 5′-AAAATGGCAGTGCGTTTAG-3′ and reverse 5′-TTTGAAGGCAGTCTGTCGTA-3′; c-myc, forward 5′-AATGAAAAGGCCCCCAAGGTAGTTATCC-3′ and reverse 5′-GTCGTTTCCGCAACAAGTCCTCTTC-3′; CD44, forward 5′-AGAAGGTGTGGGCAGAAGAA-3′ and reverse 5′-AAATGCACCATTTCCTGAGA-3′; Vimentin, forward 5′-TGTCCAAATCGATGTGGATGTTTC-3′ and reverse 5′-TTGTACCATTCTTCTGCCTCCTG-3′; Slug, forward 5′-TGTTGCAGTGAGGGCAAGAA-3′ and reverse 5′-GACCCTGGTTGCTTCAAGGA-3′. GAPDH (forward: 5′-AACGGGAAGCTTGTCATCAATGGAAA-3′, reverse: 5′-GCATCAGCAGAGGGGGCAGAG-3′) served as an internal control. Experiments were repeated three times in duplicates. Relative gene expression was calculated using the 2^-ΔΔct^ method.

### Cell proliferation and cell cycle assays

Cell proliferation was measured using a Cell Counting Kit-8 (CCK-8) (Dojindo Molecular Technologies, Kumamoto, Japan). Control cells or cells stably transfected with sh-CXCR4 or negative control were seeded into 96-well plates at 2000 cells per well and incubated overnight with or without CXCL12 (R&D, MN, USA) at 100 ng/ml. Viability of cells were measured using a Cell Counting Kit-8. Briefly, 10 μl of CCK-8 solution was added to each well after 1, 2, 3, 4 and 5 days for proliferation measurement, respectively. In viable cells, WST-8 was metabolized producing a chromogen that was detected at 450 nm using a Spectra Max M2 spectrophotometer (Molecular Devices, Sunnyvale, California, USA).

For cell cycle analysis, transfected cells were cultured for 24 h, collected, fixed into 70% ethanol at -20°C for 24 h, stained with 50 μg/ml propidium iodide (Kaiji, Nanjing, China) and analyzed with a FACS Calibur (Epics XL-4; Beckman Coulter, Brea, California, USA).

### Colony formation assay

A quantity of 500 cells transfected with either shCXCR4 or negative control were cultured in 6-well plates with or without CXCL12 for 2 weeks in regular culture medium. Colonies with more than 50 cells per colony were counted, fixed with methanol for 15 min, and stained with hematoxylin and eosin (H&E). All the experiments were performed in triplicate wells and repeated at least three times.

### β-Catenin/Tcf transcription reporter assay

Briefly, 1 × 10^5^ cells were seeded each well in a 24-well plate before transfection with the construct of TOPflash or FOPflash reporter plasmid (Millipore, Billerica, MA, USA). TOPflash comprised three copies of the Tcf/Lef sites upstream of a thymidine kinase (TK) promoter and the Firefly luciferase gene. FOPflash comprised three mutated copies of Tcf/Lef sites and was used as a control for measuring nonspecific activation of the reporter. All transfections were performed using 0.8 μg of TOPflash or FOPflash plasmid and 2 μl of Lipofectamine 2000. To normalize the transfection efficiency in reporter assays, the cells were co-transfected with 0.02 μg of an internal control reporter plasmid, containing Renilla reniformis luciferase driven by the TK promoter. Twenty four hours after transfection, the luciferase assay was performed with the Dual Luciferase Assay System kit (Promega Corp., Madison, WI, USA). Relative luciferase activity was reported as the fold induction after normalization for transfection efficiency.

### Wound healing and matrigel invasion assays

Cells transfected with negative control or shCXCR4 were seeded in 6-well plates and cultured. Upon reaching appropriate confluence, cells were serum starved for 24 h, and then the cell layer was scratched with a sterile plastic tip, immediately washed twice with PBS, and cultured in serum free 1640 medium with or without CXCL12 at 37°C in a humidified incubator with 5% CO_2_. At 24 h, the plates were photographed under a microscope.

For invasion assay, cells were re-suspended in serum-free medium and seeded in the top chambers of Matrigel-coated (invasion) chambers (24-well insert, 8 μm pore, Corning Costar Corp., Cambridge, MA, USA) at the concentration of 2 × 10^5^ per 200 μl medium. The lower chambers were filled with 0.5 ml of normal culture medium with or without CXCL12 (100 ng/ml). After 24 h, the cells on the upper surface of the membrane were removed using cotton tips, and cells that migrated to the lower surface were fixed in 4% paraformaldehyde for 15 min at room temperature, stained with hematoxylin and eosin (H&E), and counted under the light microscopy.

### Western blotting

Whole cell extracts were prepared in lysis buffer as described previously [32]. The cell lysates were separated by electrophoresis in 10% SDS-polyacrylamide gels, transferred to nitrocellulose membranes, blocked in 5% nonfat milk, and incubated with primary antibodies against CXCR4 (1:1000 dilution, Abcam), phospho-CXCR4 (1:1000 dilution, Abcam), β-catenin (1:1000 dilution, Abcam), Vimentin (1:1000 dilution, Abcam), MMP-9 (1:1000 dilution, Abcam), and β-actin (1:5000 dilution; Abcam) at 4°C overnight. After incubation with corresponding peroxidase-conjugated secondary antibodies (1:5000 dilution, Abcam), protein bands were detected using an enhanced chemiluminescence reagent (Sigma, Ronkonkoma, New York, USA).

### Tumorigenicity assay in nude mice

Five to six-week-old male nude mice used in the studies were purchased from the Institute of Zoology, Chinese Academy of Sciences (Beijing, China). After 4 days of acclimatization, a total of 2 × 10^6^ IHCC cells stably transfected with either sh-CXCR4 or negative control were injected subcutaneously into either side of the groin of each mouse (left: negative control cell, right: sh-CXCR4 for group A, and inversely for group B). Each group contained 3 mice. The mice were killed on the 28th day after injection. The mice were manipulated according to the guidelines approved by the Shandong University Institutional Animal Care and Use Committee (IACUC).

### Statistical analysis

The data are presented as percentages of control ± SEM or means ± SEM from multiple experiments. The statistical significance between groups was determined using the Student’s t-test. Overall survival was counted from the first day of surgery to the date of death or the last follow-up visit and the estimated value was calculated by the Kaplan-Meier method and compared between groups via the log-rank test. SPSS version 12 (SPSS Inc. Chicago, IL, USA) software was used for all data analyses.

## Results

### Association of CXCR4 expression level with IHCC cancers

**Figure 1:**
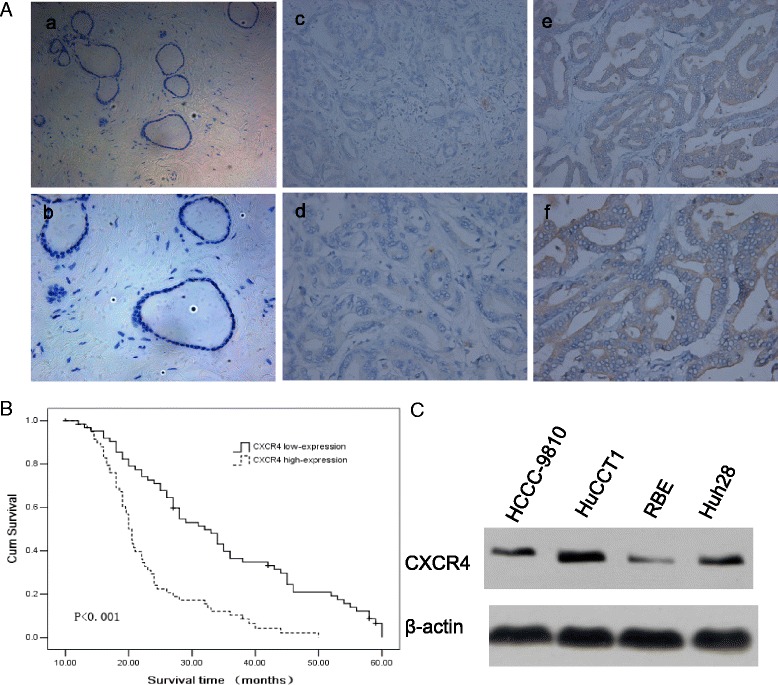
**The expression of CXCR4 on intrahepatic cholangiocarcinoma (IHCC). (A)** Immunohistochemistry staining of CXCR4 on IHCC tissues, ×200: a, c, e; ×400: b, d, f. negative control (a, b), low expression (c, d), high expression (e, f); **(B)** Kaplan-Meier survival analysis according to cytoplasmic CXCR4 expression in 122 patients with IHCC (log-rank test), P < 0.001 and the median survival time of the CXCR4 high and low expression groups was 20.0 (95% CI: 19.03-21.0) and 31.0 (95% CI: 25.6-36.4) months, respectively; **(C)** Western blot analysis revealed the CXCR4 expression for 4 IHCC cells (HCCC-9810, HuCCT1, RBE and Huh28). β-actin was used as a loading control.

Immunohistochemistry staining results showed that CXCR4 expression was detected in the cytoplasm of most IHCC cells but not in the adjacent non-tumorous tissues. The representative results are shown in Figure 1A. A total of 122 IHCC cancer patients (60 with high CXCR4 expression and 62 with low CXCR4 expression) were included in the analysis. The demographic distributions are shown in Table 1. CXCR4 expression distribution was not significantly different in age, sex, differentiation degree, and tumor size groups. However, there were more high CXCR4 expression samples among patients with vascular invasion or lymph node metastasis (63.6 and 66.7%, respectively) than in the non-vascular invasion or lymph node metastasis groups (32.1 and 21.3%; P = 0.001 and P < 0.001 for the two groups, respectively). Moreover, the proportion of high CXCR4 expression samples increased with the increased TNM stages from 13.6% in I/II stage to 72.1% in IV group (P < 0.001). In addition, the distribution of T stage showed that the percentage of high expression tumors rose significantly with increasing T stage (P < 0.001). Table 2 shows that there was no difference in the CXCR4 expression between primary IHCC and metastasized lymph nodes, implying CXCR4 expression was not affected by the different microenvironment in these sites. These data indicated that CXCR4 expression was significantly correlated with vascular invasion, lymph node metastasis, and the tumor node metastasis stages, all of which are characteristics of tumor progression and metastasis. In addition, a Kaplan-Meier analysis showed that CXCR4 was a powerful prognostic factor for overall survival (P < 0.001) and the median survival time of the high, low CXCR4 expression groups was 20.0 (95% CI: 19.03-21.0) and 31.0 (95% CI: 25.6-36.4) months, respectively (Figure 1B). *In vitro*, CXCR4 expression was detected in all four IHCC cell lines and the HuCCT-1 cell line had the highest expression, which was therefore used in the experiments described in this study (Figure 1C).

**Table 1:** **Relationship between expression of CXCR4 and clinicopathological features in IHCC**

	**CXCR4**					**χ** ^**2**^	***P*** **value**
	**Case**	**High**		**Low**			
	**n**	**n**	**%**	**n**	**%**		
Total	122	60	50.8	62	49.2		
Sex						0.688	0.447
Male	81	42	51.9	39	48.1		
Female	41	18	43.9	23	56.1		
Age						1.625	0.254
≥60	80	36	45	44	55		
<60	42	24	57.1	18	42.9		
Degree of Differentiation						3.673	0.155
Well	16	5	31.3	11	68.7		
Moderate	69	33	47.8	36	52.2		
Poorly	37	22	59.4	15	40.6		
Tumor size						0.265	0.709
<=4.0 cm	46	24	52.1	22	47.9		
>4.0 cm	76	36	47.4	40	52.6		
Vascular invasion						12.022	0.001
Present	66	42	63.6	24	36.4		
Absent	56	18	32.1	38	67.9		
CA199						6.664	0.011
≤35KU/L	53	19	34.6	34	65.4		
>35KU/L	69	41	60	28	40		
Lymph node metastasis						25.231	<0.001
No	47	10	21.3	37	78.7		
Yes	75	50	66.7	25	33.3		
TNM stage						20.443	<0.001
I and II	22	3	13.6	19	86.4		
III	57	26	45.6	31	54.4		
IV	43	31	72.1	12	27.9		
T stage						15.937	<0.001
T1-T2	48	17	35.4	31	64.6		
T3	34	13	32.4	21	67.6		
T4	40	30	75	10	25

**Table 2:** **Expression of CXCR4 in human intrahepatic cholangiocarcinomas and lymph node metastasis**

	**Case n**	**CXCR4 expression**		***P*** **value**
		**High**	**Low**	
		**n** **(%)**	**n** **(%)**	
Primary intrahepatic cholangiocarcinomas	122			
With nodal metastases	75	50 (66.7)	25(33.3)	<0.001
Without nodal metastasis	47	10 (21.3)	37(78.7)	
Intrahepatic carcinomas with nodal metastasis				
Primary IHCC	75	50(66.7)	25(33.3)	0.038
Matched lymph node metastases	75	62(82.7)	13(17.3)	

### The establishment of shCXCR4

**Figure 2:**
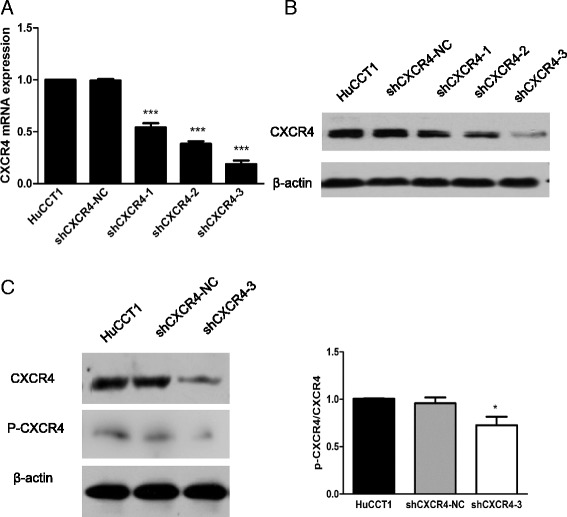
**Establishment of stable CXCR4 knockdown. (A)** QT–PCR analysis of CXCR4 expression after the transfection of different CXCR4 shRNA expression vectors: shCXCR4-1, shCXCR4-2, shCXCR4-3, shCXCR4-NC (negative control) and HuCCT1, ***P < 0.001 compared with shCXCR4-NC cells; **(B)** Western blot with CXCR4 antibody of different transfected IHCC cells. β-actin was used as a loading control; **(C)** Western blot showed the different expressions2 of CXCR4 and phosphorylated CXCR4 on Serine 339 (P339-CXCR4), together with the p-CXCR4/total CXCR4 ratio (*P < 0.05) on transfected cells. β-actin was used as a loading control.

Knockdown of transcripts using shRNA is a powerful tool to study gene function. To study the long-term growth pattern of IHCC cells *in vitro*, we constructed lenti-shCXCR4-1, -shCXCR4-2, -shCXCR4-3 and -shCXCR4-NC (negative control vector). QT–PCR analysis showed that compared with lenti-shCXCR4-NC cells, the CXCR4 mRNA expression was inhibited up to 70% in lenti-shCXCR4-2 and -3-transfected cell lines, particularly in lenti-shCXCR4-3 cells (P < 0.001), which was employed in the following experiments (Figure 2A). Moreover, the CXCR4 protein level was also downregulated significantly in lenti-shCXCR4 compared with lenti-shCXCR4-NC cells (Figure 2B). Notably, the decrease of CXCR4 phosphorylation at serine 339 was observed in lenti-shCXCR4 cells (Figure 2C).

### Abrogation of CXCR4 inhibits *in vitro* IHCC cell proliferation, cell cycle, and colony-forming ability, and *in vivo* tumorigenicity

**Figure 3:**
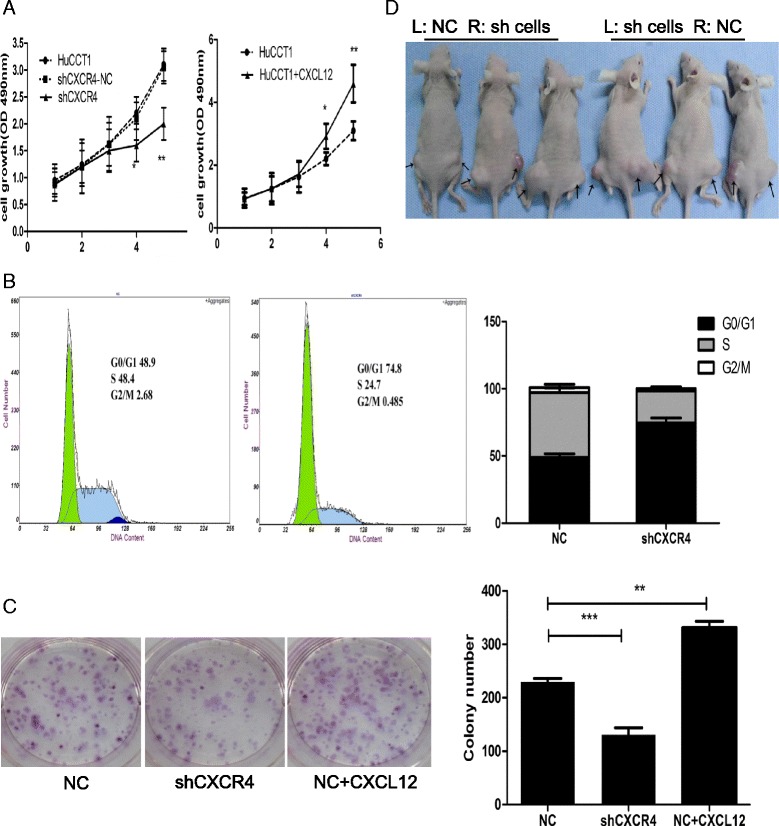
**The influence of CXCR4 knockdown on the cell phenotype. (A)** CCK-8 assay was analyzed for shCXCR4, shCXCR4-NC, HuCCT1 and CXCL12 (100 ng/ml) stimulated shCXCR4-NC on days 1, 2, 3, 4, and 5; **(B)** Distribution of cell cycle phases was demonstrated by flow cytometric analysis of shCXCR4 and shCXCR4-NC cells; **(C)** Colony assay was assessed to evaluate the cell colony formation ability. The count number of the colony was shown in the diagram; **(D)** The tumor formation ability of shCXCR4 and shCXCR4-NC cells *in vivo*. *P < 0.05, ** P < 0.01, ***P < 0.001 compared with shCXCR4-NC cells.

To confirm the inhibitory effect of CXCR4 on cell growth, cells stably transfected with lenti-shCXCR4-3 or lenti-shCXCR4-NC were cultured. CCK-8 assay showed that shCXCR4-3 cells grew slower than shCXCR4-NC cells. Upon treatment by the CXCR4 ligand CXCL12, IHCC cell growth was accelerated. The difference was significant on day 4 (P < 0.05) and day 5 (P < 0.01). However, there was no difference between HuCCT-1 cells and shCXCR4-NC cells (Figure 3A). Analysis of cell cycle distribution by flow cytometry demonstrated a prolonged and prominent delay in progression from G0 to G1 phase (48.9 vs 74.8%) together with a reduction at both S phase (48.4 vs 24.7%) and G2-M phase (2.68 vs 0.49%). To explore the effect of CXCR4 knockdown on tumorigenesis *in vitro*, we performed colony formation assay. The data showed that shCXCR4-3 decreased the colony formation of IHCC cells (P < 0.01), while CXCR4 ligand CXCL12 increased it (P < 0.001) (Figure 3C). To explore the effect of CXCR4 knockdown on tumorigenesis *in vivo*, shCXCR4-3 and shCXCR4-NC cells were injected into either side of BALB/c nude mice subcutaneously. As shown in Figure 3D, shCXCR4-3 inhibited the tumor formation of IHCC cells *in vivo*.

### CXCR4 knockdown inhibits Wnt activity, Wnt downstream genes, and the invasion-related genes

**Figure 4:**
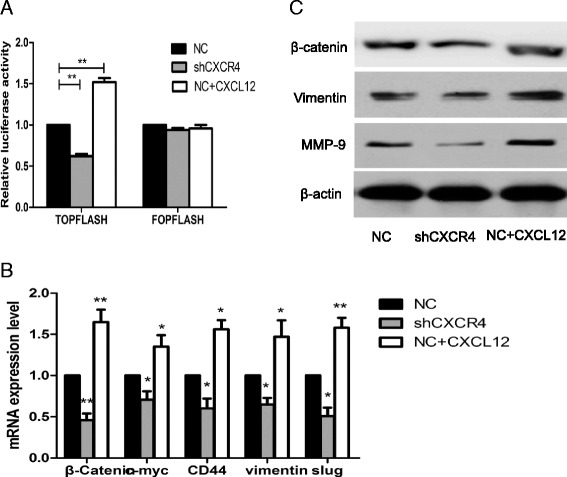
**The influence of CXCR4 knockdown on Wnt target genes and invasion**-**related genes.** **(A)** The β-Catenin/Tcf transcription reporter assay. Normalized with control reporter plasmid, the relative luciferase activity was demonstrated. **P < 0.01 compared with NC cells; **(B)** QT–PCR was applied to examine the change of Wnt downstream target genes expression including β-catenin, c-myc, CD44, Vimentin and Slug by the 2^-∆∆Ct^ method. shCXCR4 inhibited the expression of these genes, while CXCL12 enhanced their expression. *P < 0.05, **P < 0.01 compared with NC cells; **(C)** Western blot analysis was used to detect β-catenin, Vimentin and MMP-9 protein expression. β-actin was used as a loading control.

As an important pathway for gastrointestinal cancer development, the Wnt/β-catenin pathway and tumor invasion-associated genes have attracted great attention. The β-catenin/Tcf transcription reporter assay has been recognized as an important method for assessing Wnt pathway activity. Because TOPflash has three TCF-binding sites, it can be applied to measure the activation of the canonical Wnt pathway. Our data showed that compared with the shCXCR4-NC cells, the CXCR4 knockdown cells exhibited decreased TOPflash activity (P < 0.01) with FOPflash activity unchanged. Activation of CXCR4 with its ligand, CXCL12 enhanced TOPflash activity significantly (P < 0.01) but had no impact on FOPflash activity (Figure 4A). Moreover, QT–PCR analysis showed that Wnt target genes such as β-catenin, c-myc, and CD44 were decreased in shCXCR4-3 cells but increased when activated by CXCL12 (Figure 4B). Meanwhile, the inhibition of CXCR4 resulted in decreased expression of invasion-related genes Slug, Vimentin and MMP-9 and activation of CXCR4 increased the expression of these genes (Figure 4B and C). These findings suggested that the inhibitory effect of CXCR4 in IHCC cells was mediated, at least partially, through the canonical Wnt pathway.

### CXCR4 knockdown decreases the invasion and migration of IHCC cancer cells

**Figure 5:**
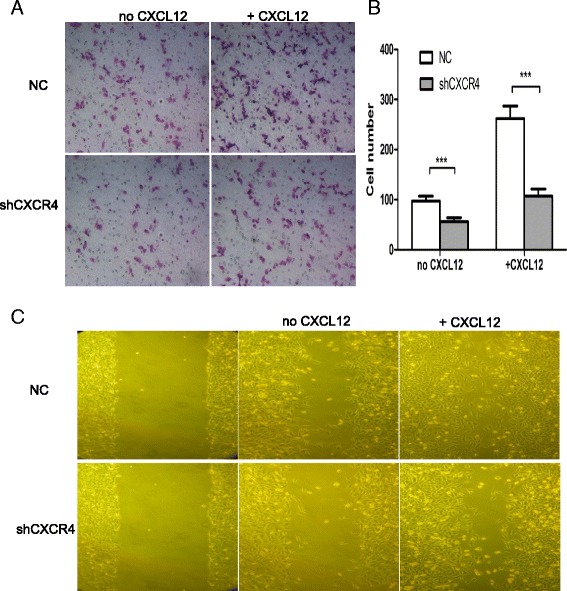
**The abrogation of CXCR4 inhibits the invasion and migration ability of IHCC cells. (A)** Representative staining figures: shCXCR4 and negative control invading cells with and without CXCL12 stimulation (100 ng/ml); **(B)** the diagram of the count analysis, ***P < 0.001 compared with NC cells; **(C)** Wound healing assay of shCXCR4 and negative control cells with or without CXCL12 stimulation (100 ng/ml).

To determine the effect of CXCR4 on cancer cell invasion and migration, Matrigel invasion and wound healing assays were performed. Representative staining results are shown in Figure 5. The data demonstrated that compared with the control cells, the migration and invasion ability of shCXCR4-transfected cancer cells were inhibited (P < 0.001) and when activated by CXCL12, shCXCR4 cells still showed a less obvious increase in both invasion and migration (P < 0.001), showing the essential role of CXCR4 in IHCC cell migration and invasion, even in CXCL12 activated cancer cells.

## Discussion

Compared with other malignancies, IHCC is generally characterized by strong proliferation, invasion, and early metastasis. Many factors such as adhesion molecules, proteases, cytokines, and chemokine are involved in these processes. CXCR4 and CXCL12 play an essential role in tumor growth, metastasis, and cancer cell-microenvironment interaction. CXCR4 has been known to be overexpressed in more than 20 human tumor types [13-18], and CXCR4 antagonists inhibit tumor growth in multiple experimental orthotopic [33,34], subcutaneous human xenografts [35,36], and transgenic mouse models [37]. Preclinical cancer models have revealed that directed metastasis of cancer cells is mediated by CXCR4 activation and migration of cancer cells is towards CXCL12 expressing organs [14,35,38] while targeting CXCR4 impairs the spread of cancer cells and development of metastasis [34,37,38]. Moreover, high levels of CXCL12 expressed by cancer cells and tumor-associated stromal cells directly stimulate the proliferation and invasiveness of breast cancer cells in the autocrine and paracrine manners [19]. High CXCL12 levels in the tumor attract CXCR4-positive inflammatory, vascular and stromal cells into the tumor mass, where they will eventually support the tumor growth by secreting growth factors, cytokines, chemokines, and pro-angiogenic factors [19,39]. In addition, CXCR4 positive cancer cells can be recruited to CXCL12-rich mesenchymal stroma niches. This recruitment mimics the homing of normal stem cells to the bone marrow [39,40], and cancer cells homed to bone marrow reside in a microenvironment that protects them in a CXCR4-dependent manner from chemotherapy [41]. In this study, we demonstrated that the overall survival rate of IHCC patients with high CXCR4 expression is significantly lower than those with low CXCR4 expression. Elevated CXCR4 expression is related to vascular invasion, lymph node metastasis, and the TNM stages. This is similar to previous reports that CXCR4 may be a useful marker for cancer progression [6,42,43]. We also found that CXCR4 shRNA not only significantly reduced the expression of CXCR4, but also notably decreased phosphorylation of CXCR4 at serine 339. Considering the findings that the phosphorylation of CXCR4 at serine 339 may be a way to activate CXCR4 on the cells [44], our data further confirmed that CXCR4 shRNA could effectively inhibit CXCR4 function in IHCC cancers.

Tumorigenesis is the result of cell cycle disorganization, leading to uncontrolled cell proliferation and cancer progression. In this study, we have demonstrated that the blockade of CXCR4 can decrease IHCC cancer cell growth and cell cycle by prolonging the G0–G1 cycle and reducing the G2 and S phases, and inhibit tumorigenesis both *in vitro* and *in vivo*. The Wnt/β-catenin pathway plays a major role in intrahepatic cholangiocarcinoma cell growth, metastasis, and cancer susceptibility [4,28-31]. Dysregulation of β-catenin and other Wnt components leads to activation of Wnt target genes, including c-myc, cyclin D1, and MMP-9 [45-47], and the enhancement of tumor formation [48]. Our data showed the TOPflash luciferase activity was sharply decreased by the inhibition of CXCR4 whereas FOPflash luciferase activity was nearly unchanged in the β-catenin/Tcf assay. Moreover, the expression of Wnt target genes, including β-catenin, c-myc, and MMP-9, was markedly decreased, suggesting that the TCF-binding activity could be effectively inhibited by CXCR4 knockdown, which may suppress theWnt/β-catenin signaling and Wnt target genes expression.

Next, we analyzed the expression of invasion-related genes Vimentin and Slug. These two genes are typical mesenchymal markers associated with the EMT process, which may influence carcinoma metastasis [49-54]. Our data showed that Vimentin and Slug were downregulated in CXCR4 knockdown cells, together with the decreased ability of invasion and migration as shown in transwell and wound healing assays. This is consistent with the previous report that CXCR4 could influence EMT formation and cancer invasion [31,55-60]. However, an intriguing phenomenon was also observed in the clinical trial of plerixafor (a CXCR4 inhibitor) as a combined treatment with intensive chemotherapy in heavily pre-treated relapse AML patients [61]. In the phase II study of 46 patients, a two-fold mobilization in leukemic blasts into the peripheral circulation was found, which was in modest correlation with CXCR4 expression. Furthermore, in a recent phase I study of another CXCR4 inhibitor LY2510924 for advanced cancer [62], the circulating tumor cell (CTC) counts were included as one of the study endpoints in addition to safety, pharmacokinetics, efficacy, and pharmacodynamics. In some (7/42) patients the CTC numbers were increased after the treatment with the CXCR4 inhibitor. Although the significance of these studies are inconclusive due to small sample sizes, these intriguing observations should prompt to investigate the mobilizing effects of CXCR4 inhibition in tumors in more details.

## Conclusions

In conclusion, the present study has shown that high CXCR4 expression is associated with metastasis and a poor clinical outcome of IHCC. CXCR4 appears to influence IHCC cell phenotype via the canonical Wnt pathway. Future *in vivo* studies will be conducted with clinical available CXCR4 inhibitors like AMD3100 or LY2510924, and data from these experiments could result in faster changes of treatment for patients with IHCC.

## Abbreviations

CXCR4: CXC chemokine receptor type 4
CXCL12: CXC chemokine ligand-12
shRNA: Short hairpin RNA
IHCC: Intrahepatic cholangiocarcinoma
EMT: Epithelial-to-mesenchymal transition
CTC: Circulating tumor cell
